# Poly (A)-specific ribonuclease deficiency impacts oogenesis in zebrafish

**DOI:** 10.1038/s41598-023-37226-6

**Published:** 2023-06-20

**Authors:** Dechamma Pandyanda Nanjappa, Hanna De Saffel, Krithika Kalladka, Srividya Arjuna, Nishith Babu, Kishan Prasad, Patrick Sips, Anirban Chakraborty

**Affiliations:** 1 Division of Molecular Genetics and Cancer, Nitte University Centre for Science Education & Research, NITTE (Deemed to be University), Deralakatte, Mangaluru, 575018 India; 2 Department of Biomolecular Medicine, Ghent University, Ghent, Belgium; 3 Department of Pathology, KS Hegde Medical Academy, NITTE (Deemed to be University), Deralakatte, Mangaluru, 575018 India

**Keywords:** Cell biology, Developmental biology, Genetics, Molecular biology

## Abstract

Poly (A)-specific ribonuclease (PARN) is the most important 3′–5′exonuclease involved in the process of deadenylation, the removal of poly (A) tails of mRNAs. Although PARN is primarily known for its role in mRNA stability, recent studies suggest several other functions of PARN including a role in telomere biology, non-coding RNA maturation, trimming of miRNAs, ribosome biogenesis and TP53 function. Moreover, PARN expression is de-regulated in many cancers, including solid tumours and hematopoietic malignancies. To better understand the in vivo role of PARN, we used a zebrafish model to study the physiological consequences of Parn loss-of-function. Exon 19 of the gene, which partially codes for the RNA binding domain of the protein, was targeted for CRISPR-Cas9-directed genome editing. Contrary to the expectations, no developmental defects were observed in the zebrafish with a *parn* nonsense mutation. Intriguingly, the *parn* null mutants were viable and fertile, but turned out to only develop into males. Histological analysis of the gonads in the mutants and their wild type siblings revealed a defective maturation of gonadal cells in the *parn* null mutants. The results of this study highlight yet another emerging function of Parn, i.e., its role in oogenesis.

## Introduction

In eukaryotic cells, degradation of messenger RNA (mRNA) by shortening of poly (A) tail (deadenylation) is a rate-limiting step that regulates mRNA turnover^1^. The poly (A)-specific ribonuclease (PARN), a deadenylase, is considered the most important enzyme involved in the process of removal of adenosine residues from poly (A) tail of mRNA in a 3′–5′ direction^2,3^. PARN, a 74 kDa polypeptide, was first identified from HeLa cells in 1991 and it belongs to DEDD superfamily of exonucleases^2,4^.

Although the primary function of PARN is to regulate the turnover of mRNAs, recent studies indicate that PARN has multiple other roles in the cell besides its canonical function in mRNA stability^5^. One of the recently discovered functions of PARN is its role in telomere maintenance. PARN processes oligoadenylated tails of H/ACA box snoRNAs^6^, a component of the telomerase RNA component (TERC), and these processed snoRNAs post-transcriptionally modify (pseudo uridylation) nascent rRNA to generate mature rRNA. In accordance with this function, PARN has been shown to localize in the nucleolus^7^, where it was found to be associated with pre-40S particles, suggesting a direct role in ribosome biogenesis. Also, PARN has been identified as one of the exonucleases involved in the processing of the 3'-end of the human 18S rRNA^8^. The unexpected discovery of *PARN* as a candidate gene mutated in inherited bone marrow failure syndromes highlights its role in hematopoiesis^9^. Indeed, it has been shown that dysregulation of mRNA adenylation can impair hematopoiesis in mice^10^ and zebrafish^11^. Considering the fact that PARN plays a role in three important processes in a cell, namely telomere maintenance, mRNA stability, and ribosome biogenesis, it is imperative that an integrated approach (animal model) is followed to systematically examine the consequences of PARN deficiency. A previous study reported the generation of *Parn* knock-out (KO) mouse model by using CRISPR/Cas9 technology^12^. While the heterozygous *Parn* KO mice were viable and displayed defects in 18S rRNA processing, the homozygous *Parn* KO mice were embryonic lethal^9,12^. Given that biallelic *PARN* mutations have been reported in patients^9,12–14^, it seems that *Parn* KO mice are not suitable animal models for studying the effects of PARN deficiency.

Maternal mRNAs support early embryonic development and are usually destabilized upon the activation of zygotic transcription. An earlier study in *Xenopus* has proposed that PARN is involved in maternal mRNA silencing during oocyte maturation and embryogenesis^15^. Here we report the generation of *parn* null mutant zebrafish that are embryonic viable and grew up as fertile adults. We show that PARN deficiency does not interfere with the embryonic development but impacts oogenesis in zebrafish, indicating a role in sex determination and gonadal maturation.

## Results

### *parn* is expressed ubiquitously during early embryogenesis

In order to detect and localize the endogenous expression of *parn* during zebrafish embryogenesis, whole-mount in situ hybridization (WISH) was performed using DIG-labelled antisense probes against *parn*. As shown in Supplementary Fig. 1, during segmentation stage (10–24hpf), *parn* expresses ubiquitously in zebrafish and the expression of *parn* becomes slightly more localised, with higher expression levels in the head and eye and to some extent in the tail region of the embryo beyond 48hpf. This suggests that the expression is ubiquitous during the early developmental stages in zebrafish.

### Generation of *parn* mutant zebrafish

**Figure 1:**
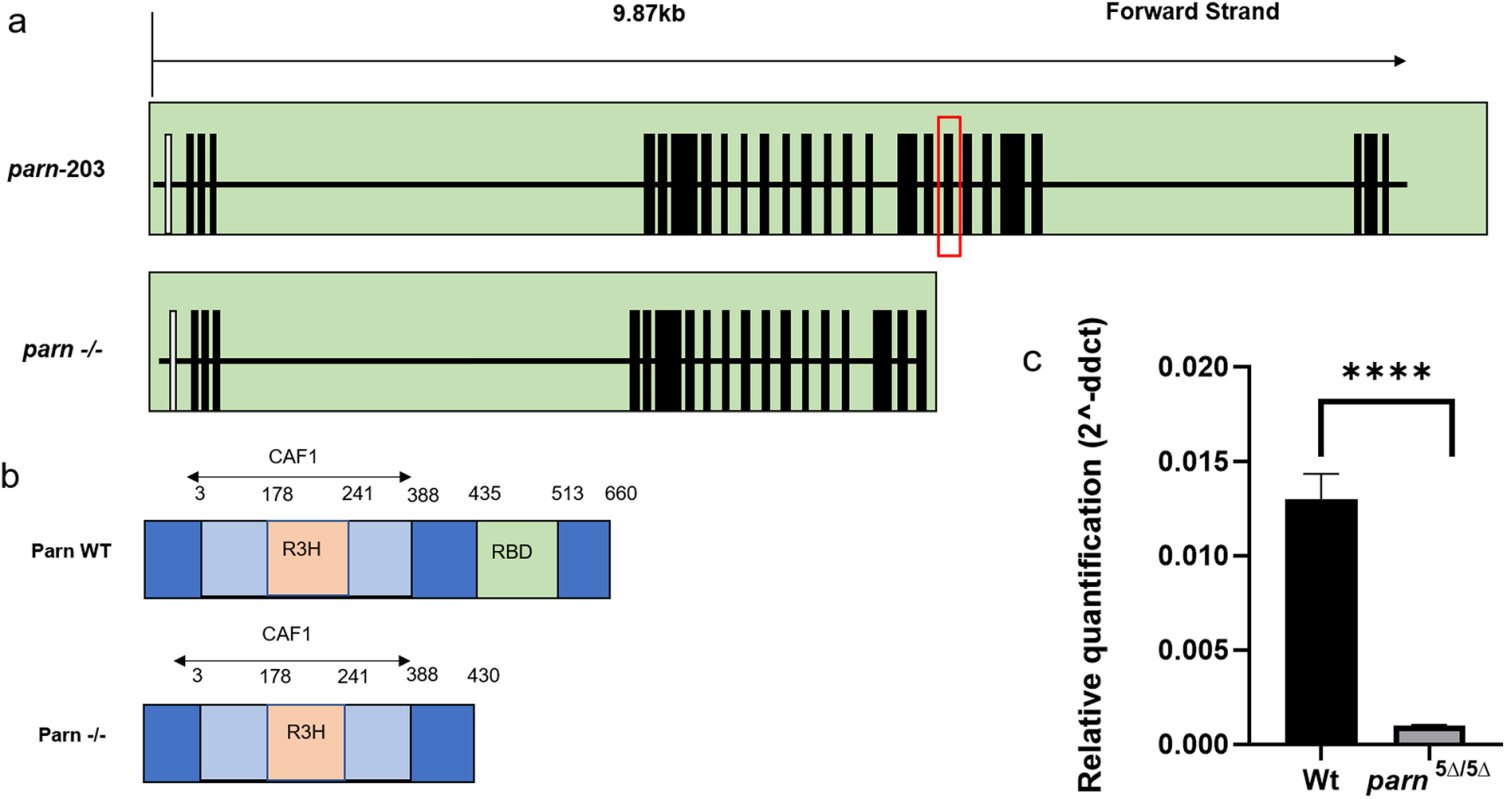
Generation of *parn*-KO zebrafish. (**a**) Pictorial representation of all the exons of *parn* and highlight of the target region. (**b**) shows the different domains (CAF1-Chromatin Assembly Factor 1 complex; R3H- Arginine and Histidine; RBD-RNA Binding Domain) of Parn wildtype and Parn *-/-.* (**c**) The graph shows the mRNA level of *parn* homozygous mutant ((*parn* ^5∆/ 5∆^) and wildtype (age-1.5 years), *p* value < 0.0001 was considered statistically significant. The error bar represents the standard deviation (SD), the results were analysed using GraphPad prism 8.4.3.

To understand the physiological consequences of a Parn loss-of-function in zebrafish, mutants were generated using the CRISPR/Cas9 gene editing tool, targeting exon 19 (Fig. 1a). Parn consists of three domains, the catalytic nuclease domain, the R3H domain and the RRM domain (Fig. 1b). The targeted exon 19 region codes for the RRM domain and previous studies have shown that the PARN binds to the poly (A) tail through the RRM domain^16^. Human and zebrafish Parn share 66% homology in their amino acid sequences, with a high degree of conservation in the RRM domain (Supplementary Fig. 2). Screening of founders by sanger sequencing of the *parn* sequence in CRISPR/Cas9 injected zebrafish revealed four different deletions in the targeted region (Supplementary Table 1). The 5 bp deletion (CCTGA), which resulted in the generation of a premature stop codon at amino acid position 430, c.1290_1294del; p.Tyr430* (here after referred to as *parn* ^5∆^ ), encoding the RNA binding domain, was selected for further analysis. The details of Sanger sequencing showing the 5 bp deletion, in exon19 of *parn* gene are shown in Supplementary Fig. 3. In order to check the transcript level of *parn* in the homozygous mutants (F1), qPCR was performed for quantifying the mRNA levels of *parn* and compared with wild type adults. As expected, in homozygous mutants the mRNA level was significantly reduced compared to that in the wildtype (Fig. 1c).

### *parn* mutant zebrafish are phenotypically indistinguishable from wild type siblings at embryonic stages

**Figure 2:**
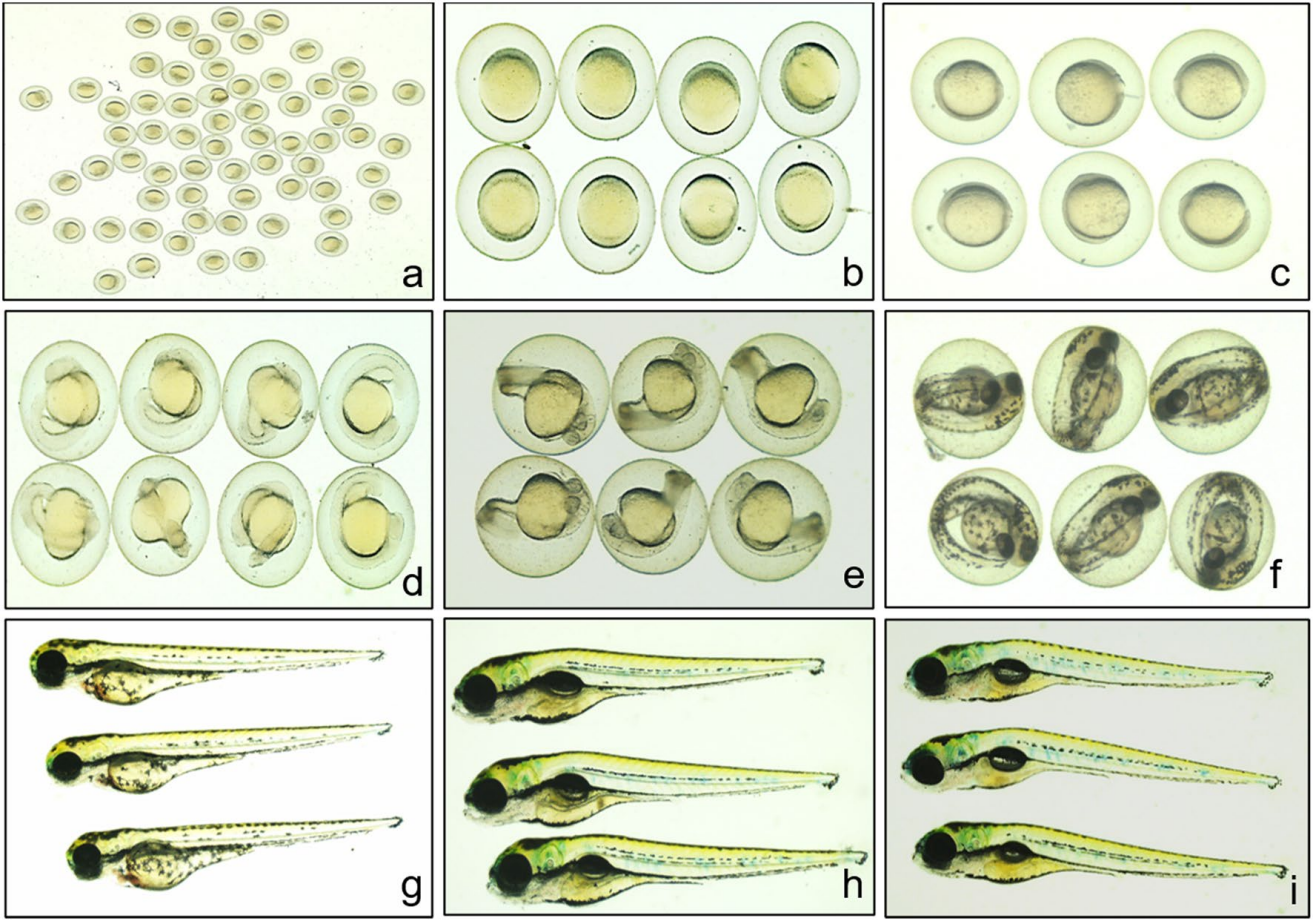
Representative images of embryos obtained from a *parn* heterozygous (*parn* ^5∆/+^*)* cross at 0hpf (**a**), 6hpf (**b**), 10hpf (**c**), 18hpf (**d**), 24hpf (**e**), 48hpf (**f**), 72hpf (**g**), 96hpf (**h**) and 120hpf (**i**) to show that all were phenotypical identical. All images were captured using Leica S9D, Camera MC190.

After having confirmed the loss of 5 bp del in *parn*, the early morphologic development of *parn* mutant zebrafish was monitored at 6hpf, 10hpf, 18hpf, 24hpf, 48hpf, 72hpf, 96hpf and 120hpf (Fig. 2) through phenotypic observation of a clutch of embryos obtained from an in cross of *parn* heterozygotes. There were no morphological anomalies in any of the embryos in the clutch, suggesting the lack of developmental phenotypes in the heterozygous and homozygous mutant zebrafish. In order to further confirm this observation, various combinations of male and female adults with different zygosity patterns (*parn* ^5∆ /5∆^ , *parn* ^5∆ /+^, *parn* ^+*/*+^) were crossed and representative clutches from each cross were morphologically evaluated for developmental phenotypes and other assays including survivability, heart rate, hatchability and swimming behaviour. The schematics of the different crosses are shown in Supplementary Fig. 4.

**Figure 3:**
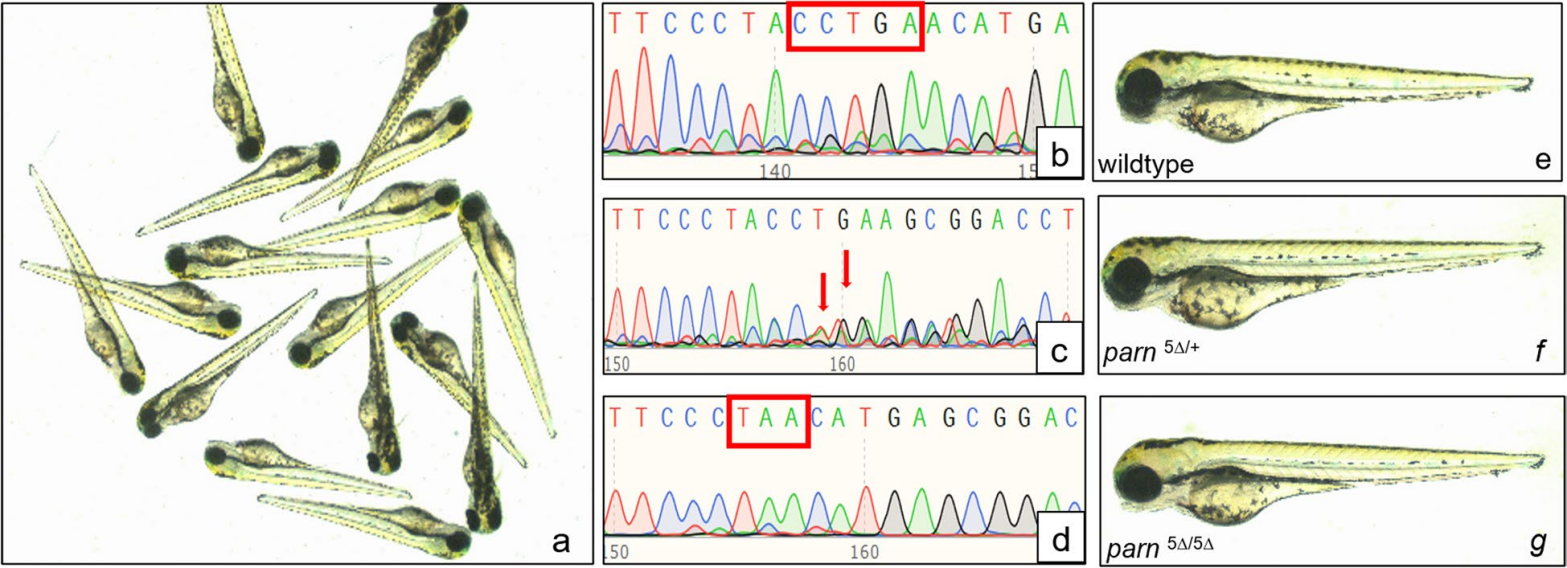
(**a**) Representative images of embryos obtained from a *parn* heterozygous cross at day 3. (**b**–**d**) shows the electropherogram of wildtype, heterozygous (*parn* ^5∆/+^) and homozygous (*parn* ^5∆/5∆^) mutant embryos respectively. The region highlighted in ‘b’ shows 5 bases ‘CCTGA’ intact in wildtype. In ‘c’ the arrows represent the rearrangement of bases due to loss of 5 bases ‘CCTGA’ in one allele. The highlighted region in ‘d’ shows the stop codon (TAA) that arises due to loss of 5 bases ‘CCTGA’ in both the allele. (**e**), (**f**), (**g**) corresponds to embryos at day 3 for wildtype, heterozygous and homozygous mutant respectively with no morphological difference. All images were captured using Leica S9D, Camera MC190. Polypeak parser (yosttools.genetics.utah.edu/PolyPeakParser/) software was used to analyse the sequencing data.

None of the crosses gave any clutch of embryos that were morphologically distinguishable from the siblings, indicating that the *parn* homozygous and heterozygous mutant zebrafish are phenotypically indistinguishable from wild type siblings and are not lethal at embryonic stages. To further confirm the mutation, genotyping was performed at day 3 using single embryo from a clutch of heterozygous cross and representative images of different zygosity are shown in Fig. 3.

#### Assessment of survival rate

The offspring of different crosses, were observed for five days with change of media twice a day. There was no significant difference in survival until day 5. The percentage of surviving embryos in each clutch ranged from 83 to 97% across all five experimental groups (Supplementary Fig. 5).

#### Assessment of heart rate

The resting heart rate was assessed across all test groups and a control as mentioned above at the age of 48hpf. The values obtained are mean of three independent observations (Supplementary Table 2) and no significant changes were observed in any of the clutches indicating no effect of Parn loss-of-function on cardiac function in the embryos.

#### Assessment of hatching rate

The zebrafish embryos hatch and leave the chorion usually by 72 h post fertilization. There was no difference in hatching rate of any of the embryos in the clutches observed at day 2 (48hpf) and day 3 (72hpf), suggesting no defect in this developmental milestone (Supplementary Fig. 6).

### Juvenile *parn* mutant zebrafish are normal but develop exclusively as males

**Figure 4:**
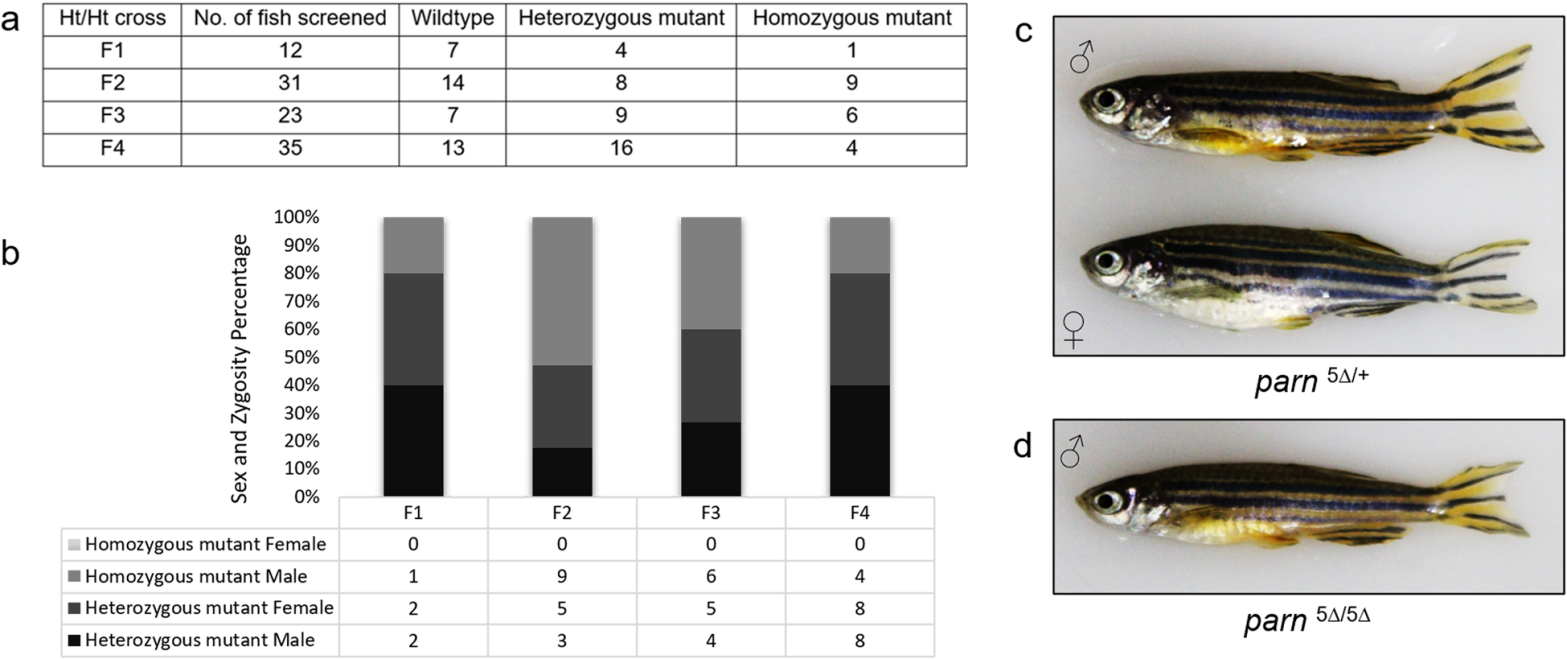
(**a**) Number of mutants obtained across 4 generation. (**b**) Shows the sex and the zygosity percentage obtained across 4 generation. (**c**, **d**) Representative images of adult *parn* ^5∆/+^ ♂, *parn* ^5∆/+^ ♀ and *parn* ^5∆/ 5∆^ ♂ showing no morphological difference (age-1.5 years). The images (**c**) and (**d**) were captured using a DSLR camera (Canon EOS1300D).

The offspring of *parn* ^5∆ /+^ ♀ x *parn* ^5∆ /+^ ♂, *parn* ^5∆ /+^ ♀ x *parn* ^5∆ /5∆^ were grown to juvenile stages and adulthood. None of the offspring obtained from both these crosses showed any morphological abnormalities at juvenile stages. Representative images of the embryos obtained from a heterozygous in-cross are shown in Supplementary Fig. 7. The adult heterozygous mutants were in crossed and their genotype was determined once the fish attained sexual maturity. The number of males and females were also noted across four generations, while the *parn* heterozygous mutant zebrafish developed in equal proportions into males and females, the *parn* null mutant zebrafish only developed into males indicating a defective gonadal development in the null mutant zebrafish (Fig. 4).

### Adult *parn* null mutant zebrafish males are fertile

**Figure 5:**
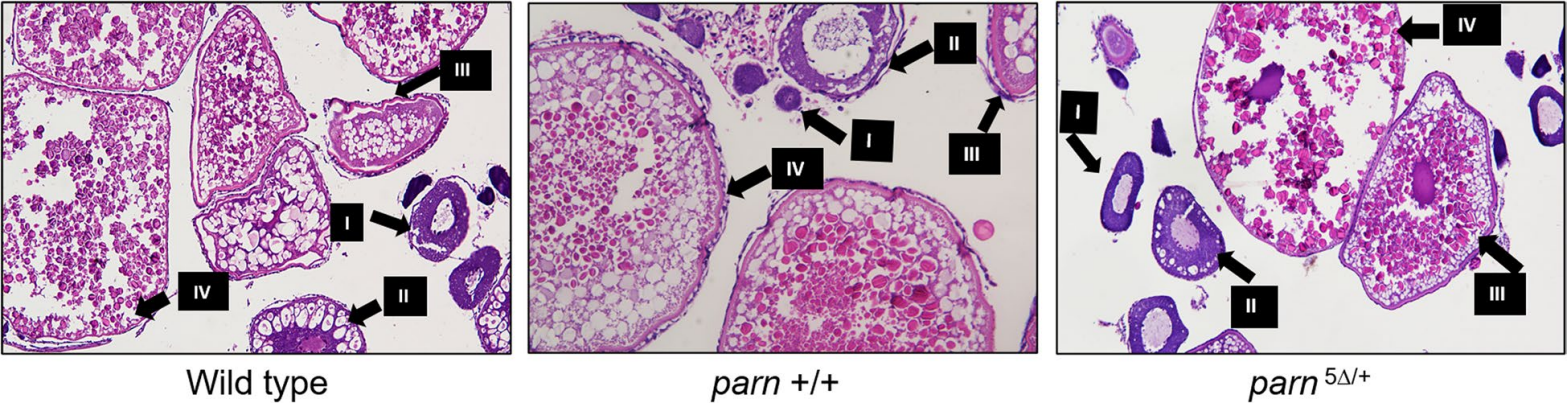
Representative sections of ovary tissue stained with hematoxylin and eosin, from wildtype ♀, *parn* + / + ♀ obtained from *parn* ^5∆/+^ cross and *parn* ^5∆/+^ ♀ obtained from *parn* ^5∆/+^ cross. The oocytes are shown with arrows and the numbers represent oocytes at corresponding stages of development, Pre-vitellogenic stages indicated as I and II; vitellogenic stage indicated as III and post vitellogenic stage indicated as IV (age-1.5 years). The images were captured using Olympus BX53, Camera DP74, Bright field. ht corresponds to the *parn* heterozygous mutants (*parn* ^5∆/+^).

**Figure 6:**
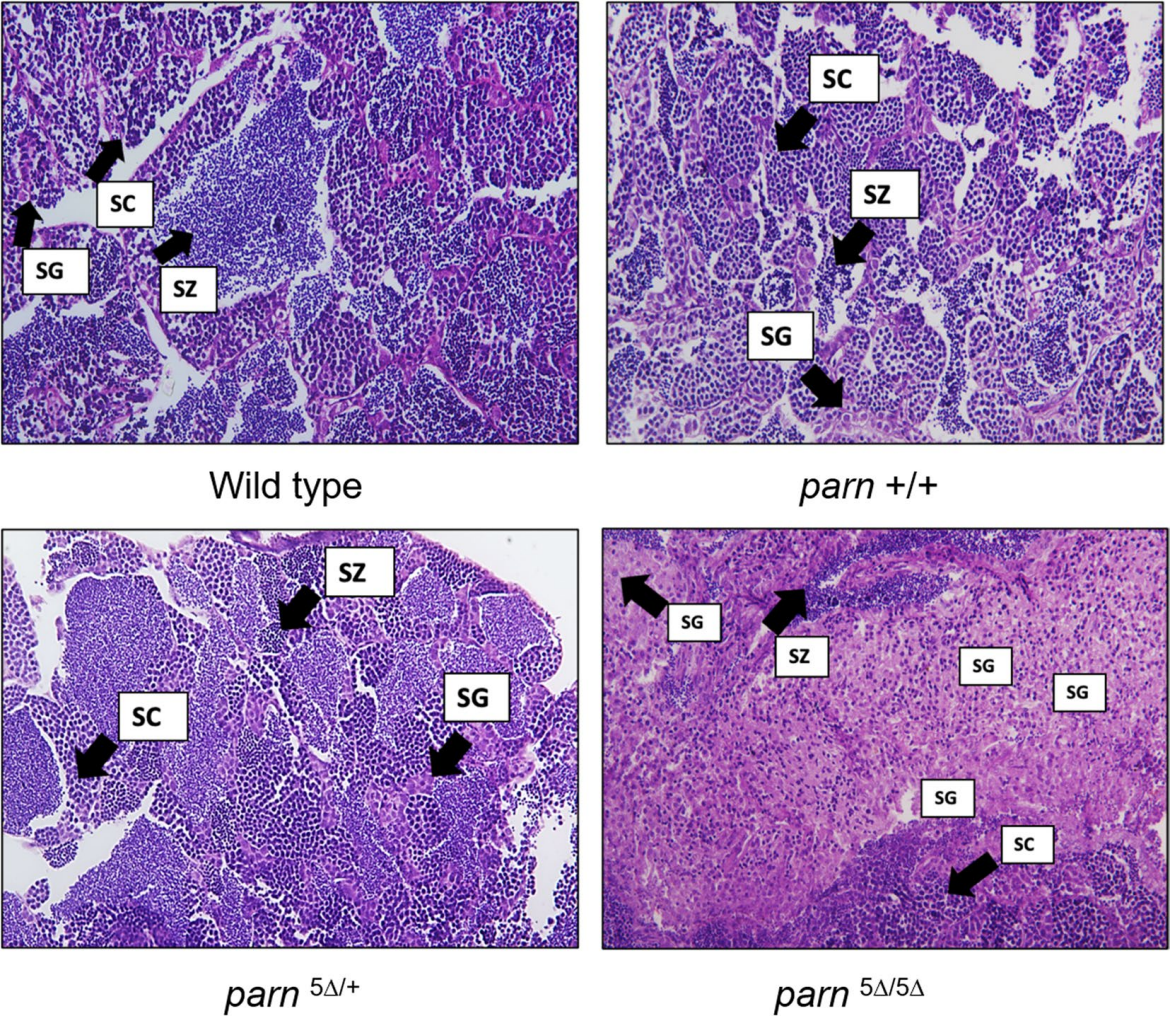
Representative sections of testis tissue stained with haematoxylin and eosin, from wildtype ♂, *PARN* + / + ♂ obtained from *parn* ^5∆/+^ , *parn* ^5∆/+^ ♂ obtained from *parn* ^5∆/+^ cross and *parn* ^5∆/ 5∆^ obtained from *parn* ^5∆/+^ cross. SC: Spermatocytes, SG: Spermatogonia, SZ: Spermatozoa (age-1.5 years). The images were captured using Olympus BX53, Camera DP74, Bright field. ht corresponds to the *parn* heterozygous mutants and hm corresponds to the *parn* homozygous mutants.

Since the *parn* null mutant zebrafish developed only as males, a number of zebrafish obtained from *parn* mutation background were screened for this gonadal phenotype and genotyped for *parn*. Histological analysis of the gonads in the mutant zebrafish and their wild type siblings revealed a defective maturation of gonadal cells in the *parn* null mutants. Interestingly, there was no obvious morphological difference in oogenesis between females obtained from a wildtype cross and females (both wildtype and *parn* heterozygous mutants) obtained from *parn* heterozygous (*parn* ^5∆ /+^ ♀ x ♂) cross, with the presence of all stages of oocytes in the ovaries (Fig. 5). The histological analysis of the testis in these mutant zebrafish showed no difference in spermatogenesis in *parn* wild type, heterozygous and homozygous zebrafish. However, the null mutant zebrafish showed an increase in the number of spermatogonia and decreased number of spermatocytes compared to wild type and heterozygous ones (Fig. 6). Assessment of fertility was done by crossing *parn* ^5∆ /+^ female with *parn* ^5∆ /5∆^ male and *parn* ^5∆ /5∆^ male with AB line females. In all the trials, the fish spawned naturally resulting in fertilised and healthy embryos, suggesting no effect on fertility of the male zebrafish due to loss of Parn.

### Endogenous *parn* shows very high expression in ovary

**Figure 7:**
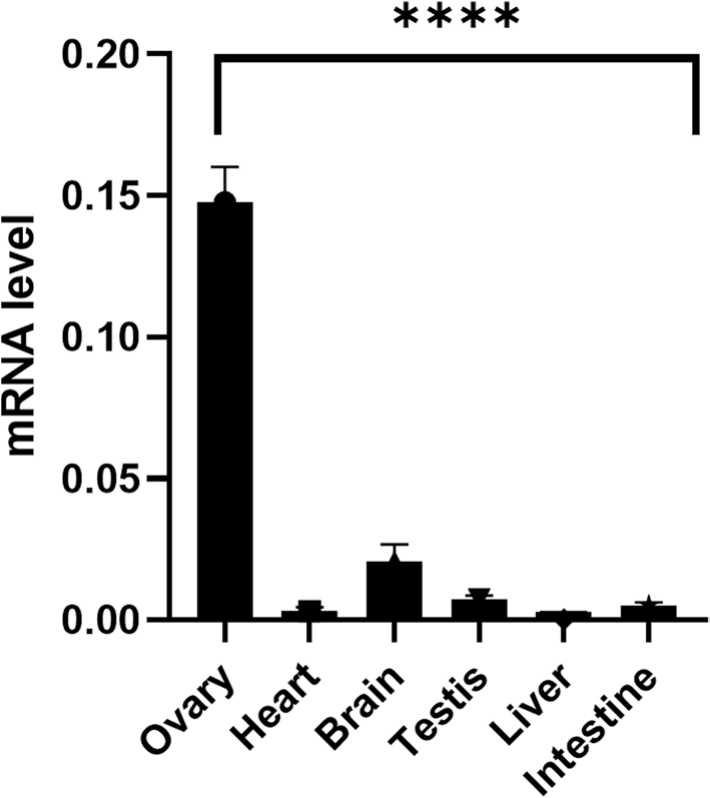
The graph shows the basal mRNA level of *parn* in different organs of adult zebrafish. Of all the organs, ovary show higher mRNA transcript when compared to heart, testis, liver, intestine, kidney and brain. (Age-1.5 years), *p* value < 0.0001 was considered statistically significant. The error bar represents the standard error mean (SEM), the results were analysed using GraphPad prism 8.4.3.

In order to further understand the reason for the specific phenotype in *parn* null mutant zebrafish, the endogenous transcript levels of *parn* in major organs in wildtype adult zebrafish was analysed. *parn* was found to be expressed in all the major organs including brain, heart, testis, liver, intestine, kidney, and ovary in concordance with the fact that it is an important deadenylase, and it is expected to be ubiquitously present in all the organs. However, the expression of *parn* in the ovary was very high compared to other organs, which corroborates the all-male phenotype observed in the *parn* null mutant zebrafish (Fig. 7).

## Discussion

Conventionally, PARN is known for its role in mRNA decay pathway through its ability in removing the poly (A) tail from the 3′ end of mature mRNA. However, recent studies show several other functions of PARN including a role in ribosome biogenesis, stability of noncoding RNAs^17^, TP53 regulation^18,19^ and telomere maintenance^13,14^. It is also found to be mutated in several diseases associated with telomere dysfunction and in genetic conditions involving bone marrow failures^9,20,21^. Also, our previous studies showed that *PARN* is consistently downregulated in non-small cell lung cancers^22^ and *PARN* depletion in cell lines results in differential and cell-specific alterations in the expression of various tumor suppressor and oncogene mRNAs^23^. Given the fact that PARN is now considered a protein that has multiple roles in cells besides just mRNA stock clearing^5^, development of an alternative animal model to systematically examine the effects of complete loss of PARN was very much warranted as the previous attempt to generate *Parn*-null mice was unsuccessful^12^. In this study, we used CRISPR/Cas9 gene editing to develop *parn* -deficient zebrafish. Contrary to our expectations, *parn* null mutant zebrafish were not only embryonic viable but also grew up as fertile adults. However, the null mutant zebrafish turned out to be exclusively males, suggesting an effect on oogenesis. An earlier study looked at transient downregulation of *parn* in zebrafish through morpholinos and the authors reported that *parn* morphants were anaemic and leukopenic^9^. However, in our study we did not see these phenotypes in *parn* CRISPR/Cas9-generated mutant zebrafish. Morpholinos have been widely used in the zebrafish model system as important antisense agents. Despite its wide popularity, morpholinos are often associated with off-target effects and possibly artefactual phenotypes, including pericardial edema, body curvature, and also anemia, commonly observed in morpholino-induced gene knockdown. On the other hand, a number of reports in zebrafish have indicated that CRISPR/Cas9-generated mutants do not always display the same phenotypes when compared to the results seen with morpholinos for the same genes^24,25^. A possible explanation for these observations is the concept of genetic compensation, where the generation of a mutant allele results in the upregulation of genes compensating the function of the mutant gene, through regulation of the non-sense mediated decay machinery^26,27^. Since the mutated *parn* allele generated in our study is prone to non-sense mediated decay, the possibility exists that genetic compensation mechanisms are activated in the *parn* mutant zebrafish.

All the *parn* null mutant zebrafish were phenotypically male at sexual maturity whereas their heterozygous siblings were both male and female and this was observed in at least four generations in our study. These results may indicate that Parn is required for oogenesis. Indeed, an earlier study in *Xenopus laevis,* showed that PARN is involved in deadenylation of maternal mRNAs and oocyte maturation^15^. Thus, our observation seems to corroborate the results obtained in *Xenopus*.

Interestingly, *brca2* null mutants*,* just like *parn* null mutant zebrafish, develop as only males but are infertile^28^. In adult zebrafish, *brca2* is expressed in both developing oocytes and in mature oocytes. However, in testis it is expressed only in spermatocytes and developing spermatocytes, but not in mature sperm. Thus, *brca2* is important for ovarian development. Also, mutants involved in DNA repair (*atm-/-)* lead to male-only phenotypes^29^ while the *fancl-/-* were all male and fertile^30^. Both *atm* and *fancl* are associated with DNA repair pathways and they play a role in sex determination in zebrafish through their involvement in the maturation and differentiation of primordial germ cells (PGC). Taken together, the above phenotypes in zebrafish mutant further validates our observation on the role of Parn in oogenesis. Indeed, our results on endogenous expression of *parn* in adult zebrafish organs showed highest level in ovaries, further confirming the tissue-specific phenotype that was observed in *parn* depleted zebrafish.

The role of TP53 in mediating apoptosis of the primordial germ cells has been suggested as the possible mechanism in all-male phenotype in *atm* and *fancl* null zebrafish. Interestingly, we have shown that downregulation of *PARN* in cell lines reduces TP53 transcript levels^23^. Thus, it would be interesting to see if the TP53 pathway is responsible for impaired oogenesis in *parn* null mutants as seen in *atm* and *fancl* null mutants.

Maternal mRNAs support early embryonic development are usually destabilized upon the activation of zygotic transcription. An earlier study in Xenopus has shown that *PARN* is involved in the inactivation of maternal mRNAs^15^. Therefore, an alternative hypothesis, which needs to be validated, is that improper maternal mRNA degradation due to *parn* deficiency delayed the process of zygotic transcription and that led to a deregulation in the key events involved in the differentiation of gonads in *parn* null mutant zebrafish.

Nevertheless, the data presented here clearly imply that Parn plays a crucial role in sex differentiation in zebrafish. It would therefore be interesting to explore *parn* as a candidate gene in disorders of sexual development, more specifically congenital conditions where the development of gonads and anatomical sex is atypical.

## Materials and methods

### Zebrafish husbandry and rearing

Adult zebrafish (AB line) maintained at rearing facility with temperature 28 ± 0.5 °C and 14/10 h light and day dark cycle were used in the study. The fish were fed four times a day with dry feed and once with live artemia. To obtain embryos for the experiment, pairwise mating of fish was done and fertilised eggs were collected the following day.

### Whole mount in situ hybridization

Whole mount in situ hybridization was performed to detect and localize endogenous expression of *parn* during zebrafish embryogenesis. A digoxigenin-labelled antisense RNA probe against *parn* was generated by cloning a *parn* PCR product (564 bp, primer details is given in Supplementary Table 3) into pDrive vector (Qiagen, Germany). The positive colonies were checked for correct orientation and were used as templates for antisense RNA synthesis using in vitro RNA synthesis kit (Roche, USA). The endogenous expression of *parn* was checked at 10hpf, 18hpf, 24hpf, 48hpf and 72hpf in wildtype zebrafish embryos (AB line).

### sgRNA design and gBlock construct preparation

sgRNA was designed using CRISPOR (https://crispor.tefor.net/) website targeting exon 19 of RNA Binding domain of Parn. The gBlock construct that contained at the 5′- end, a random 8 bp sequence (CCGCTAGC), followed by the T7 promoter sequence (TAATACGACTCACTATA), the target-specific sequence without the adjacent PAM sequence (TACCTGAACATGAGCGGACC) and the constant region of the sgRNA (TAGTCCGTTATCAACTTGCAAAAAGTG GCACCGAGTCGGTGCTTTT) at the 3′-end was obtained from IDT (USA) and was used for sgRNA synthesis by in vitro transcription. The gBlock fragment was obtained in lyophilised form and was dissolved in RNAse free water (concentration of 10 ng/µl) upon arrival. The in vitro transcription was performed using the MEGAshortscript™ T7 Transcription Kit (Invitrogen) following the manufacturers instruction. Post transcription, the product was purified and the quality was analysed using capillary electrophoresis (BioRad, USA). The Cas9 protein (wild-type nuclease protein with nuclear localization signal) was procured from ToolGen (South Korea).

### Injection of CRISPR components and screening for mutants

1 ng Cas9 protein and 200 pg sgRNA, complexed as a ribonucleoprotein in a solution containing 300 mM KCl, were injected in approximately 100 zebrafish embryos (AB line) at 1 cell stage. At 1 day post fertilization (dpf), 15–20 embryos were harvested for DNA isolation and the region of interest containing the sgRNA target site was PCR amplified with specific primers using the KAPA HiFi HS ReadyMix (NIPPON Genetics, Tokyo, Japan). The amplified product was subjected to MiSeq deep sequencing according to Metagenomic Sequencing Library Preparation kit protocol (Illumina, San Diego, CA, USA). The deep sequencing data was analyzed using the previously reported Batch-GE pipeline^31^. MiSeq data of three independent CRISPR/Cas9 injection experiments indicated efficiencies of 65.72%, 86.15%, and 72.86% (on a total of 747, 643, and 328 read pairs respectively).

### Screening for founders

Since the F0 animals carry one or more mutation in a mosaic fashion, screening of the potential founders was done (Supplementary Table 1). For this, F0 were grown to adulthood and F1 were obtained by outcrossing the founders with wild-type zebrafish. The F1 embryos were screened for mutation by Sanger sequencing.

### Genotyping of the *parn* mutants

The fish were anesthetized using 0.016% MS-222 (Tricaine) and transferred immediately onto a petri dish using a plastic spoon. The caudal fin was clipped with a sterile blade. The clipped tail was transferred to a 50 mM NaOH solution, and denatured at 95 °C for 20 min. Next, the homogenate was neutralized by addition of 1/10 volume 1 M Tris–HCl (pH 7.0). The homogenate was used as a template for PCR to amplify a fragment containing the CRISPR/Cas9 cut site. The product was electrophoresed on an agarose gel post PCR (genotyping primer detail is given in Supplementary Table 3) and sent for Sanger sequencing to check for the presence of the mutation. For genotyping of embryos, crude DNA was extracted from the single embryo at 72hpf as per earlier protocol^32^. All the genotyping analysis were carried out using the online tool poly peak parser (http://yosttools.genetics.utah.edu/PolyPeakParser/).

### Morphological observation of *parn* mutant zebrafish

Morphological observation of *parn* null and heterozygous mutant zebrafish included a detailed inspection of the embryos for developmental phenotypes at 24hpf, blood circulation at 48hpf, swimming behaviour at 72hpf and organ formation at 120hpf, using light microscopy (Leica S9D, Camera MC190).

### Gonad histology of *parn* mutant zebrafish

Four groups containing wildtype (2 male, 2 female), *parn* heterozygous (2 male, 2 female), wildtype *parn* sibling (2 male, 2 female) and *parn* homozygous (4 male) were included for gonadal histology. Ovaries and testis were removed and fixed in 10% formalin. Next, the tissues were paraffin embedded, sectioned and stained with haematoxylin and eosin to determine the morphology of the gonad tissues.

### RNA isolation, cDNA synthesis and real time PCR

Total RNA was extracted from tissues (heart, ovary, testis, liver, intestine, kidney and brain) of adult wildtype zebrafish using RNeasy Minikit (QIAGEN, Germany. One µg of RNA was reverse transcribed to cDNA using a cDNA synthesis kit (Takara) and 0.5 ng was used as the template for Real-time PCR to determine the expression level of *parn* using QuantStudio3 Real Time thermocycler (Thermo Fisher Scientific, USA). The mRNA levels were estimated by the ΔΔCT method using *elf1α* as the internal control. The primer information is given in Supplementary Table 4.

### Statistical analysis

All the statistical analysis (t test, two tailed) were carried out using Microsoft Excel 2010 and GraphPad prism 8.4.3. The experiments were carried out in triplicates and *p* value < 0.0001 was considered statistically significant.

### Ethical approval

This study was approved by the Institutional Animal Ethics Committee of NGSM Institute of Pharmaceutical Sciences (NGSMIPS/IAEC/Nov-2019/154). All methods were performed following the relevant guidelines and regulations and also in accordance with ARRIVE guidelines**.**

## Supplementary Information

Supplementary Information.

## Data Availability

All data analysed/generated during this study are included in this article and its supplementary information files.

## References

[1] Chen CY, Shyu AB (2011). Mechanisms of deadenylation-dependent decay. Wiley Interdiscip. Rev. RNA.

[2] Martinez J (2000). A 54-kDa fragment of the poly (A)-specific ribonuclease is an oligomeric, processive, and cap-interacting poly (A)-specific 3′ exonuclease. J. Biol. Chem..

[3] Martinez J, Ren YG, Nilsson P, Ehrenberg M, Virtanen A (2001). The mRNA cap structure stimulates rate of poly (A) removal and amplifies processivity of degradation. J. Biol. Chem..

[4] Astrom J, Astrom A, Virtanen A (1991). In vitro deadenylation of mammalian mRNA by a HeLa cell 3'exonuclease. EMBO J..

[5] Nanjappa DP (2021). Poly (A)-specific ribonuclease (PARN): More than just “mRNA stock clearing”. Life Sci..

[6] Berndt H (2012). Maturation of mammalian H/ACA box snoRNAs: PAPD5-dependent adenylation and PARN-dependent trimming. RNA.

[7] Ishikawa H (2017). Poly (A)-specific ribonuclease regulates the processing of small-subunit rRNAs in human cells. Nucleic Acids Res..

[8] Montellese C (2017). Poly (A)-specific ribonuclease is a nuclear ribosome biogenesis factor involved in human 18S rRNA maturation. Nucleic Acids Res..

[9] Dhanraj S (2015). Bone marrow failure and developmental delay caused by mutations in poly (A)-specific ribonuclease (PARN). J. Med. Genet..

[10] Stumpo DJ (2009). Targeted disruption of Zfp36l2, encoding a CCCH tandem zinc finger RNA-binding protein, results in defective hematopoiesis. Blood.

[11] Bolli N (2011). cpsf1 is required for definitive HSC survival in zebrafish. Blood.

[12] Benyelles M (2019). Impaired telomere integrity and rRNA biogenesis in PARN-deficient patients and knock-out models. EMBO Mol. Med..

[13] Moon DH (2015). Mutations in the Poly (A)-specific ribonuclease (PARN) impair telomerase RNA 3'end maturation in dyskeratosis congenita patients. Nat. Genet..

[14] Tummala H (2015). Poly (A)-specific ribonuclease deficiency impacts telomere biology and causes dyskeratosis congenita. J. Clin. Investig..

[15] Copeland PR, Wormington M (2001). The mechanism and regulation of deadenylation: Identification and characterization of Xenopus PARN. RNA.

[16] He GJ, Zhang A, Liu WF, Yan YB (2013). Distinct roles of the R3H and RRM domains in poly(A)-specific ribonuclease structural integrity and catalysis. Biochim. Biophys. Acta..

[17] Shukla S, Parker R (2017). PARN modulates Y RNA stability and its 3′-end formation. Mol. Cell. Biol..

[18] Baquero J (2019). Nuclear Tau, p53 and Pin1 regulate PARN-mediated deadenylation and gene expression. Front. Mol. Neurosci..

[19] Shukla S, Bjerke GA, Muhlrad D, Yi R, Parker R (2019). The RNase PARN controls the levels of specific miRNAs that contribute to p53 regulation. Mol. Cell..

[20] Burris AM (2016). Hoyeraal-Hreidarsson syndrome due to PARN mutations: Fourteen years of follow-up. Pediatr. Neurol..

[21] Belaya Z (2021). Multiple bilateral hip fractures in a patient with dyskeratosis congenita caused by a novel mutation in the PARN gene. Osteoporos. Int..

[22] Babu N (2021). Expression of poly (A)-specific ribonuclease in solid tumours and haematopoietic malignancies. J. Clin. Diagn. Res..

[23] Babu N, Nanjappa DP, Nazareth S, Arjuna S, Chakraborty A (2022). PARN knockdown in cell lines results in differential and cell-specific alterations in the expression of cancer-associated mRNAs. Asian Pac. J. Cancer Prev..

[24] Novodvorsky P (2015). klf2a sh317 mutant zebrafish do not recapitulate morpholino-induced vascular and haematopoietic phenotypes. PLoS One.

[25] San B (2019). The ezh2 (sa1199) mutant zebrafish display no distinct phenotype. PLoS One.

[26] Peng J (2019). Gene redundancy and gene compensation: An updated view. J. Genet. Genomics.

[27] Rouf MA (2022). The recent advances and future perspectives of genetic compensation studies in the Zebrafish Model. Genes Dis..

[28] Shive HR (2010). brca2 in zebrafish ovarian development, spermatogenesis, and tumorigenesis. Proc. Natl. Acad. Sci..

[29] Vierstraete, J. *et al*. Atm deficient zebrafish model reveals conservation of the tumour suppressor function. In *Belgian Society for Human Genetics, 20th Annual meeting, Abstracts*. http://hdl.handle.net/1854/LU-8652925 (2020).

[30] Rodriguez-Mari A, Canestro C, BreMiller RA, Nguyen-Johnson A, Asakawa K, Kawakami K, Postlethwait JH (2010). Sex reversal in zebrafish fancl mutants is caused by Tp53-mediated germ cell apoptosis. PLoS Genet..

[31] Boel A (2016). BATCH-GE: Batch analysis of next-generation sequencing data for genome editing assessment. Sci. Rep..

[32] Dupret B, Völkel P, Follet P, Le-Bourhis X, Angrand PO (2018). Combining genotypic and phenotypic analyses on single mutant zebrafish larvae. MethodsX.

