# Supplementary material for: Poly (A)-specific ribonuclease deficiency impacts oogenesis in zebrafish
Source: Sci Rep. 2023 Jun 20;13:10026. doi: 10.1038/s41598-023-37226-6 (PMC10281955; doi:10.1038/s41598-023-37226-6)
Supplement: Supplementary file 1 — Supplementary Information. [file 41598_2023_37226_MOESM1_ESM.pdf]

Supplementary data

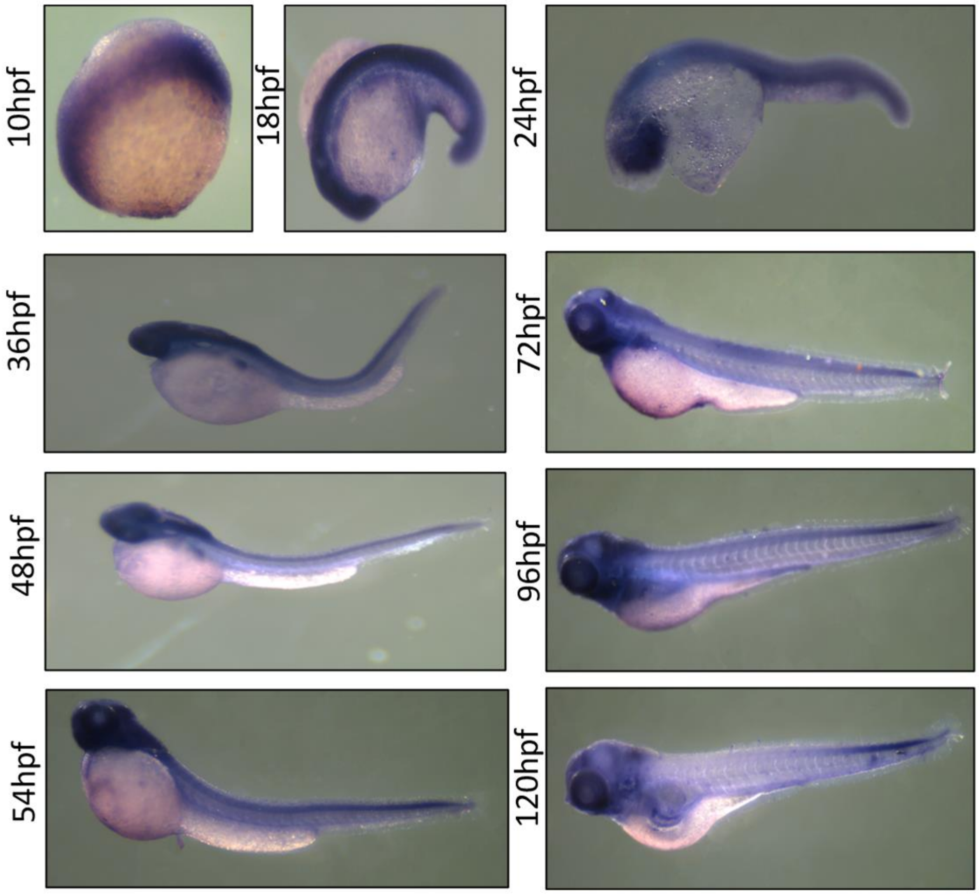

Supplementary Figure 1-The endogenous expression of *PARN* at 10hpf, 18hpf, 24hpf, 36hpf, 48hpf, 54hpf,72hpf,96hpf and 120hpf in wildtype zebrafish embryos. The expression pattern was found to be concentrated in the head, eye and tail of the embryo. hpf stands for hours post fertilization. The images were captured using a light microscope , Leica S9D, Camera MC190.

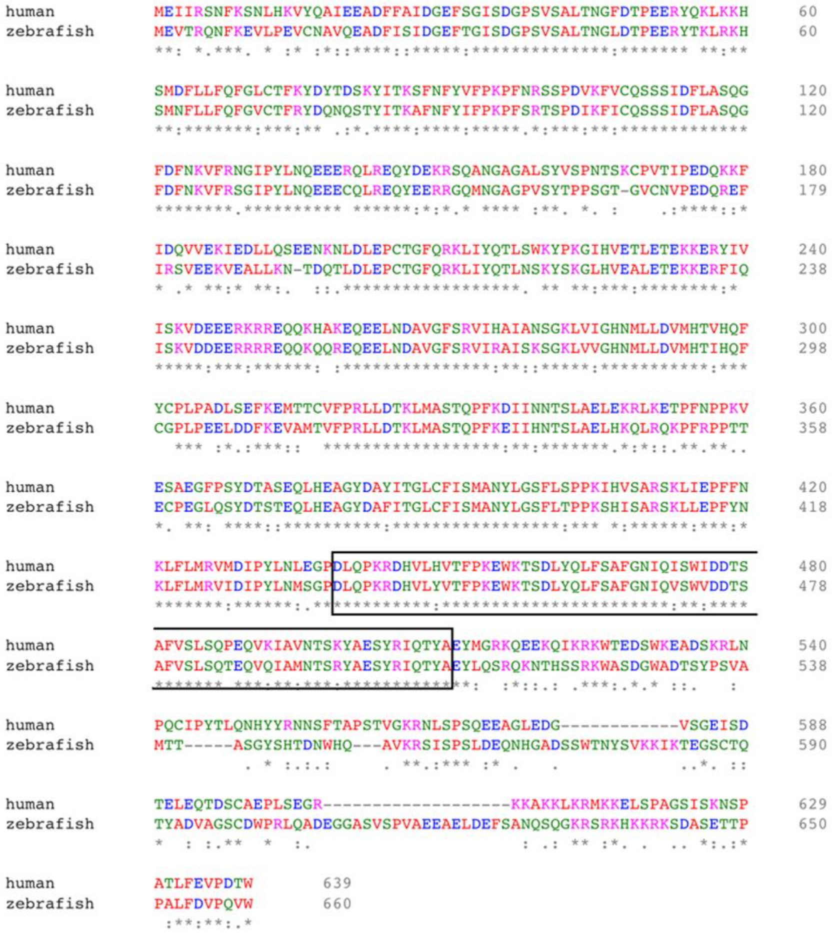

Supplementary Figure 2- Multiple sequence alignment of human and zebrafish amino acids done using clustal omega (https://www.ebi.ac.uk/Tools/msa/clustalo/) software. The overall homology between human and zebrafish amino acid sequence is 66%. The highlighted region marks the RNA Recognition motif (RRM) domain indicating the high degree of conservation where the similarity between human and zebrafish amino acid sequence is 90%. Asterisk (*) corresponds to the conserved residue, colon (:) represents the conservation of residue with similar properties, fullstop (.) are residues with low conservation properties.

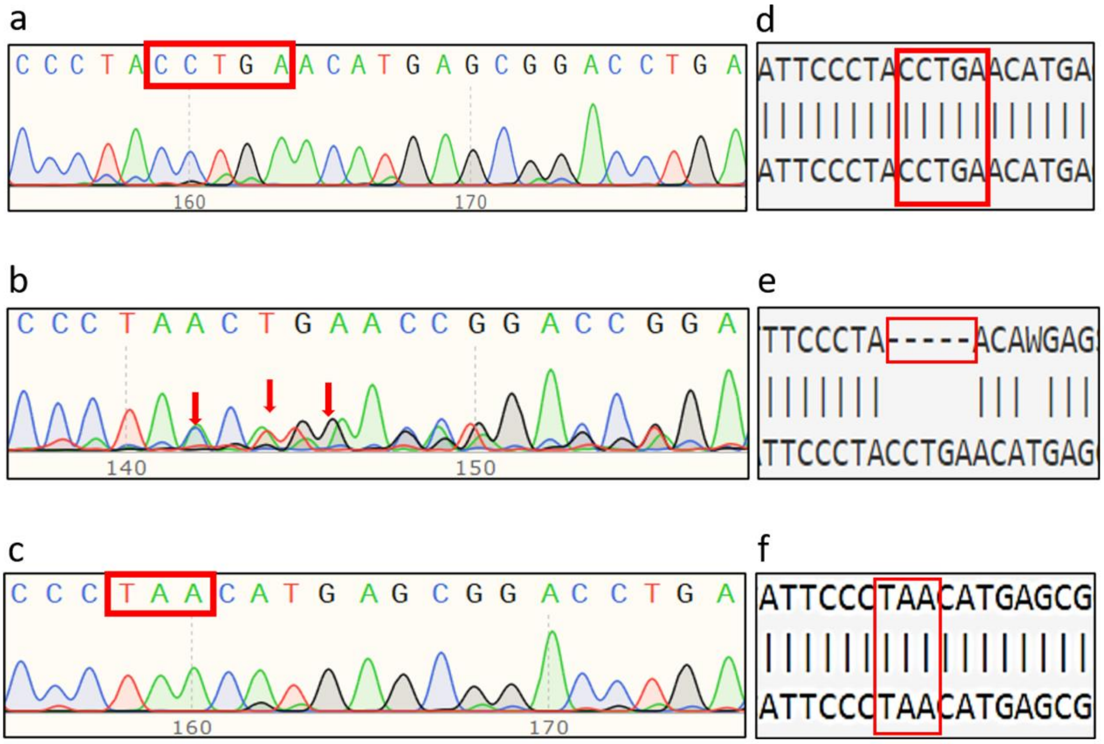

Supplementary Figure 3- Representative images showing electropherogram of *parn* 5bp deletion. (a), (b), (c) shows the electropherogram of wildtype, heterozygous and homozygous mutant respectively. (d),(e),(f) is the alignment of target sequence with reference sequence for wildtype, heterozygous and homozygous mutant respectively using the software polypeak parser (yosttools.genetics.utah.edu/PolyPeakParser/). The region highlighted in (a) and (d) shows 5 bases ‘CCTGA’ intact in wildtype. In (b) the arrows represent the rearrangement of bases due to loss of 5 bases ‘CCTGA’ in one allele which is clearly shown in (e). The highlighted region in (c) and (f) represents the stop codon (TAA) that arises due to loss of 5 bases ‘CCTGA’ in both the alleles.

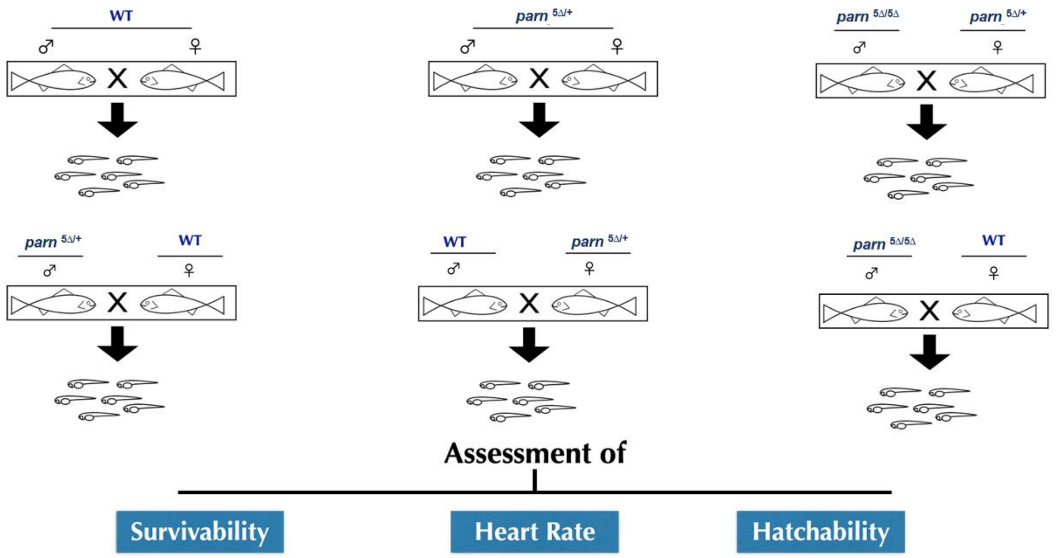

Supplementary Figure 4- Diagram of the breeding process between male and female zebrafish (WT, *parn* ht, parn hm; age 1.5 years) and the resultant representative clutches obtained from each cross, which were assessed for developmental and morphological parameters. ht corresponds to the *parn* heterozygous mutants, hm corresponds to the *parn* homzygous mutants.

a

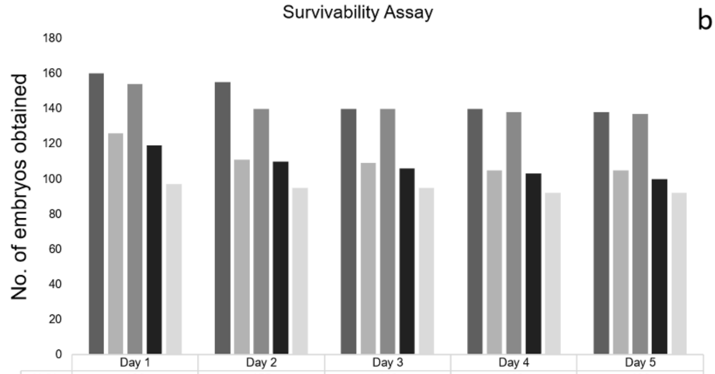

	Day 1	Day 2	Day 3	Day 4	Day 5
Ht/Ht	160	155	140	140	138
Ht/Hm	126	111	109	105	105
Ht/Wt	154	140	140	138	137
Hm/Wt	119	110	106	103	100
Wt/Wt	97	95	95	92	92

b

PARN 5bp del cross	% Surviving at Day 5
Ht ♀/ Ht♂	86%
Ht ♀/ Hm♂	83%
Ht ♀/ Wt♂	88%
Wt♀/ Hm ♂	84%
Wt ♀/ Wt♂	94%

Supplementary Figure 5- The graphs show the survivability of embryos from day 1 to day 5 and the table shows the percentage of embryos surviving at day 5, obtained from different crosses. Ht corresponds to the *parn* heterozygous (*parn* $^{5\Delta/+}$) mutants, hm corresponds to the *parn* homozygous (*parn* $^{5\Delta/5\Delta}$) mutants and wt is the wildtype. The data was calculated using Microsoft Excel 2010.

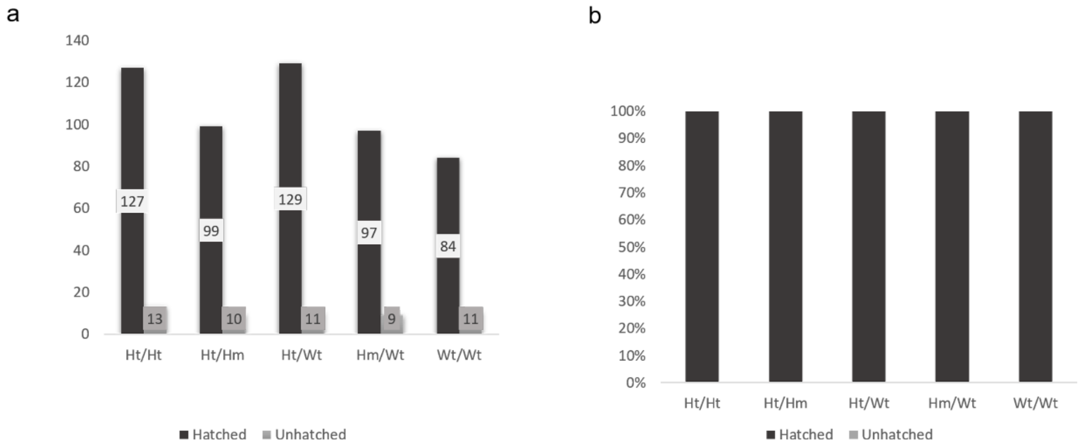

Supplementary Figure 6- Percentage of hatched and unhatched embryos obtained from crosses of males and females of different PARN genotype at day 2 (a) and day 3 (b). Ht corresponds to the *parn* heterozygous (*parn* $^{5\Delta/+}$) mutants, hm corresponds to the *parn* homozygous (*parn* $^{5\Delta/5\Delta}$) mutants and wt is the wildtype. The results were analysed using Microsoft Excel 2010.

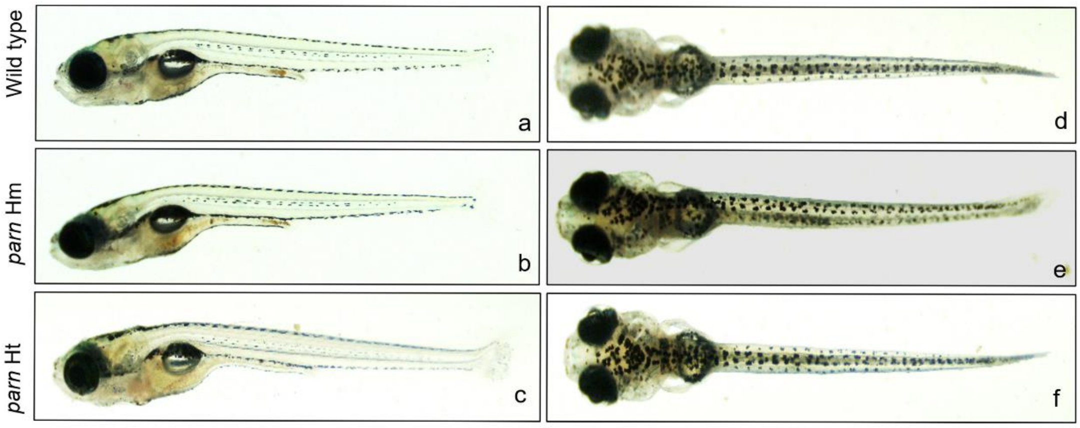

Supplementary Figure 7- Juvenile morphology-Representative image obtained from *parn* heterozygous ♀/ heterozygous ♂ (Age- 24dpf (a,b,c)- lateral view (d,e,f)- dorsal view). Ht corresponds to the *parn* heterozygous (*parn* $^{5\Delta/+}$) mutants, hm corresponds to the *parn* homozygous (*parn* $^{5\Delta/5\Delta}$) mutants and wt is the wildtype. The images were captured using Leica S9D, Camera MC190.

Supplementary Tables

Supplementary Table 1: List of deletions observed after CRISPR/Cas9 recombination

Wildtype	GGGTCATCGACATT**CCCTACCTGAACATGAGCGGACC**TGACTGTAAGAAACACACAC
5bp del	GGGTCATCGACATTCCCTA**CCTGA**ACATGAGCGGACCTGACTGTAAGAAACACACAC
6bp del	GGGTCATCGACATTCCCTAC**CTGAAC**ATGAGCGGACCTGACTGTAAGAAACACACAC
5bp del	GGGTCATCGACATTCCCTACCTGAA**CATGA**GCGGACCTGACTGTAAGAAACACACAC
1bp del	GGGTCATCGACATTCCCTAC**C**TGAACATGAGCGGACCTGACTGTAAGAAACACACAC

Supplementary Table 2: Assessment of Heart rate

PARN 5bp del cross	Trial 1	Trial 2	Trial 3	Mean
Ht ♀/ Ht♂	155	153	156	154.6667
Ht ♀/ Hm♂	157	158	151	155.3333
Ht ♀/ Wt♂	154	159	160	157.6667
Wt♀/ Hm ♂	150	157	151	152.6667
Wt ♀/ Wt♂	154	151	156	153.6667

Supplementary Table 3: Genotyping primers

PARN Probe-F	5’-AGGAGTGTGGAGGAAAAGGTT-3’	Amplicon size-564bp
PARN Probe-R	5’-AAGGCCTCCAGAGTTACGAC-3’	
PARN Geno-F	5’-GTTTCTCCTCAGGCTCTTTCCT-3’	Amplicon size-410bp
PARN Geno-R	5’-CTGAACAGCTGGTACAGGTCA-3	

Supplementary Table 4: Real time primers

PARN RT-F	5’-AGGAGTGTGGAGGAAAAGGTT-3’	Amplicon size- 156bp
PARN RT-R	5’-CTTCTCAGTCTCCAGCGTC-3’	
zelf1α RT-F	5’-GGG AAA GGA AAA GAC CCA CA -3’	Amplicon size-180bp
zelf1α RT-F	5’-AAC ACC CAG GCG TAC TTG AA -3’	
